# Chronic Pain with Neuropathic Characteristics in Diabetic Patients: A French Cross-Sectional Study

**DOI:** 10.1371/journal.pone.0074195

**Published:** 2013-09-13

**Authors:** Didier Bouhassira, Martine Letanoux, Agnès Hartemann

**Affiliations:** 1 INSERM U-987, Centre d’Evaluation et Traitement de la Douleur, CHU Ambroise Paré, APHP, Boulogne-Billancourt, France; 2 Université Versailles-Saint-Quentin, Versailles, France; 3 Centre Medical, Cherche Midi, Paris, France; 4 Service de Diabétologie, CHU Pitié-Salpétrière, Paris, France; University of Würzburg, Germany

## Abstract

**Objective:**

Our aim was to estimate the prevalence of distal chronic pain with neuropathic characteristics in patients with type 1 and type 2 diabetes mellitus and its impact on quality of life, mood, anxiety, sleep and healthcare utilization.

**Methods:**

In total, 885 patients were screened and 766 diabetic patients (38.7% with type 1 diabetes mellitus, 44.8% women, mean age: 57.2±14.9 years) were enrolled consecutively over a three-month period in this observational study by 85 diabetes specialists working in a hospital department or in private practice. All the patients completed a series of questionnaires for the detection of chronic pain (i.e. daily pain for more than three months) in the lower limbs and assessment of health-related quality of life (Medical Outcomes Short Form 12 scale), sleep disturbances (MOS sleep scale), depression and anxiety (Hospital Anxiety and Depression scale). Patients with chronic pain were also assessed with the 7-item DN4-interview questionnaire, the monofilament test and the Michigan Neuropathy Screening Instrument (MNSI).

**Results:**

The overall prevalence of chronic pain with neuropathic characteristics was 20.3% [95% CI 17.4–23.1]. The MNSI examination score suggested that pain was related to polyneuropathy, in 80.1% of these patients (89.5% of those with bilateral pain). Patients with chronic pain had a poorer quality of life and more sleep disturbances, anxiety and depression than patients without pain and the presence of neuropathic characteristics was predictive of such impairments. Only 38.6% of the patients had received appropriate treatment for neuropathic pain.

**Conclusions:**

Chronic pain with neuropathic characteristics concerns one in five diabetic patients, has a significant impact on quality of life and is not adequately managed. The close correlation between the DN4 questionnaire and MNSI results suggests that screening tools for neuropathic pain could be used in daily practice for the identification of painful diabetic polyneuropathy.

## Introduction

Sensory distal polyneuropathy is a common complication of diabetes, affecting about 50% of the patients. It may have severe complications, including foot ulcers, amputation and chronic pain [1], [2]. The prevalence of painful diabetic polyneuropathy (PDPN) remains unclear, with estimated prevalence ranging from 10 to 60% of diabetic patients, probably reflecting differences in the diagnostic criteria used in different studies [3]–[5].

Pain associated with diabetic polyneuropathy is, by definition [6], neuropathic, and thus has a number of clinical characteristics in common with other neuropathic pain syndromes related to various lesions or diseases of the nervous system [7], [8]. In the last few years, several clinical tools, in the form of simple questionnaires, have been developed and validated for the screening of neuropathic pain, for use in both clinical research and daily clinical practice [9], [10]. These tools based on the identification of specific pain qualities, rely mostly on the terms used by the patients to describe their pain (pain descriptors) and have been shown to have excellent sensitivity and specificity for the identification of neuropathic pain in various populations of patients [10]. Thus, although not specific to diabetes, these clinical tools could improve the identification of painful diabetic neuropathy.

The primary objective of this observational study was to estimate the prevalence of distal (lower limbs) chronic pain with neuropathic characteristics (identified with the DN4 questionnaire [7]) in patients with type 1 or type 2 diabetes mellitus and to assess the relationship of this pain to polyneuropathy.

Like other chronic neuropathic pain syndromes [12], painful diabetic neuropathy can significantly alter the patients’ quality of life [13]–[16]. Another goal of this study was thus to assess the impact of pain on health-related quality of life (QoL), sleep, mood and anxiety and the use of healthcare resources and analgesic treatments by patients.

## Methods

### Participants

This multicenter observational study was managed by a multidisciplinary scientific committee and was carried out in accordance with the principles of the Helsinki Declaration. The study was approved by the ethic committee, Commission de Protection des Personnes (CPP) Ile de France 1, and all patients provided written informed consent before enrollment. Patients were enrolled consecutively over a three-month period (up to 10 patients per center) by diabetes specialists working in a hospital department or in private practice. The inclusion criteria were: patient with type 1 or type 2 diabetes mellitus, over the age of 18 years, with a good understanding of French and with a HbA_1c_ determination carried out in the last four months.

### Study Design

Data were collected through standardized questionnaires. The investigators recorded patients’ demographics, diabetes characteristics, complications, treatments and cardiovascular risk factors. The patients were then asked to report any pain they were suffering in their feet and/or legs and its duration. If the patients reported chronic pain in the lower limbs, defined as daily pain for at least three months, the investigator recorded the treatments for pain received by the patient and administered the DN4 questionnaire, the monofilament test and the MNSI (Michigan Neuropathy Screening Instrument) (see below), before finally noting the cause of pain, according to their own clinical judgment.

All the patients (with and without pain) were asked to complete a series of questionnaires to assess health-related quality of life, sleep disturbances and anxiety and depression (see below). The patients with chronic pain, were asked to rate the mean intensity of pain over the previous week on a numerical rating scale (0 = no pain, 10 = worst pain possible), to specify its location, its duration and who they had consulted for this pain.

### Assessment of Neuropathic Pain and Diabetic Peripheral Neuropathy

The DN4-interview questionnaire was used to identify neuropathic characteristics of pain. This questionnaire has two questions, comprising seven items: three items relating to pain quality and four items relating to associated paresthesia/dysesthesia. Patients with a score ≥3 were considered to have pain with neuropathic characteristics [7], [11].

The 10-g Semmes-Weinstein monofilament test was performed, as described by Perkins et al. [17], [18], for the detection of a tactile hypoesthesia. A reference stimulus was first applied to the forehead or the sternum. The patients were then asked to close their eyes, and the monofilament was applied to a noncallused site on the dorsum of the big toe, just proximal to the nail bed perpendicular to the skin. This maneuver was repeated four times per foot. The stimuli were applied randomly to each foot with no null stimuli used. The test results were considered normal if the patient perceived at least 50% or more of the applications.

The MNSI [19] has two parts. The MNSI-questionnaire includes 15 “yes” or “no” questions on foot sensation, including pain, numbness and temperature sensitivity. A score ≥7 was considered suggestive of neuropathy. The MNSI examination consists of a standardized physical examination including: i) inspection of the feet for deformities, dry skin, hair or nail abnormalities, calluses or infection; ii) the detection of foot ulceration; iii) the grading of ankle reflexes; iv) a semi-quantitative assessment of sensation of vibration at the dorsum of the big toes. A score >2 (maximum score 8) was regarded as suggestive of the presence of polyneuropathy.

### Assessment of Quality of Life, Sleep Disturbances, Anxiety and Mood

The assessment of quality of life in patients with and without pain was based on the Medical Outcomes Short Form 12 scale (MOS SF-12) [20], a validated self-administered tool for measuring health status derived from the SF-36, which has been used to measure functioning and symptoms, including pain, in various chronic pain conditions, including neuropathic pain [12]. It consists of 12 questions assessing eight different domains. The scores are summarized into two summary components, corresponding to mental and physical health, for each of which the score ranges from 0 (worst possible health state) to 100 (best possible health state). Sleep was assessed with the MOS sleep scale [21], a 12-item instrument assessing six dimensions (optimal sleep/quantity of sleep, sleep adequacy, sleep disturbance, snoring, awakening from sleep with shortness of breath or headaches, somnolence). A sleep problem index summarizing information for MOS sleep items can also be calculated. This index ranges from 0 to 100, with higher score associated with larger numbers of sleep disorders. This scale has been shown to be reliable and valid for use in patients with chronic pain, including neuropathic pain [12]. Symptoms of anxiety and depression were assessed with the Hospital Anxiety and Depression scale, which includes 14 items, each assessing anxiety (7 items) or depression (7 items) and scored out of 21. Higher scores indicate higher levels of anxiety or depression [22]. The use of healthcare resources was assessed by questions concerning visits to physicians and the types of physician consulted about pain in the last 12 months (primary care physician, diabetologist, neurologist, surgeon, rheumatologist, pain specialist, other).

### Statistical Analysis

Continuous variables, which had all a normal distribution, are described as means ± SD; categorical variables are expressed as frequency distributions and percentages for the relevant subject groups, with 95% confidence intervals. Missing data were not considered in the expression of the results.

Pain intensity scores of 1 to 3 were considered to indicate mild pain, scores of 4 to 6 were considered to indicate moderate pain, and scores of 7 to 10 corresponded to severe pain.

A logistic regression model was used to identify the factors (age, sex, types of diabetes, HbA_1C_ level, diabetes complications, risk factors) potentially predictive of chronic pain with neuropathic characteristics. The variables significant in the univariate analysis, at a threshold of at least 20%, were entered into a septwise multivariate logistic regression model and the odds ratio for significant factors was calculated with a 95% confidence interval (CI). One-way analyses of variance (ANOVA) were used to compare the scores for quality of life (SF-12), sleep disorders (MOS-Sleep) and mood disorders (HADS) between the various groups of patients. A logistic regression analysis was used to identify the factors (age, sex, type of diabetes, HbA_1C_, complications, risk factors, pain intensity, DN4 score, MNSI-examination score, monofilament test) predictive of an altered SF-12, HADS or MOS-Sleep scores. Stepwise logistic regression models were used, as described above, to identify factors independently predictive of alterations of these scores. Data were analyzed with SAS version 9.1 software (SAS Institute, Cary, NC, USA).

## Results

In total, 885 patients were screened, 85 declined to participate and 34 did not meet the inclusion criteria. The clinical characteristics of the 766 consecutive patients with type 1 (38.8%) or type 2 (61.2%) diabetes included in the study are summarized in table 1. The 85 participating centers: 43% in hospital departments and 57% in private practice, were distributed nationwide but with particularly dense concentrations in “Ile de France” (i.e. the Paris area: 27%) and “Provence Alpes Côte d’Azur” (11%), consistent with the geographic distribution of French physicians.

**Table 1:** Characteristics of the patients.

	Patients with type1 diabetes (*n* = 297)	Patients with type2 diabetes (*n* = 469)	Total (*n* = 766)
Women	142 (47.8%)	201 (42.9%)	343 (44.8%)
Age (years)	48.3±16.0	62.9±10.7	57.2±14.9
Duration of diabetes (years)	20.8±12.4	13.9±9.0	16.6±11.0
HbA1c level (%)	7.9±1.3	7.6±1.4	7.7±1.3
HbA1c level ≥7%, n (%)	241 (81.1%)	291 (62.0%)	618 (70.1%)
Risk factors			
Total Cholesterol (mmol/L)	4.7±1.1	4.5±1.1	4.6±1.1
HDL-Cholesterol (mmol/L)	1.4±0.5	1.2±0.4	1.3±0.4
Triglycerides	1.1±0.8	1.7±1.2	1.5±1.1
BMI	25.4±4.7	30.6±6.0	28.6±6.0
Obesity (BMI ≥30 kg/m^2^)	47 (15.8%)	231 (49.3%)	278 (36.3%)
High Blood Pressure (>130/80 mmHg)	114 (38.3%)	315 (67.2%)	430 (56.1%)
Diabetic complications			
Diabetic retinopathy	97 (32.7%)	107 (22.8%)	204 (26.8%)
Diabetic nephropathy (proteinuria >300 mg/L)	37 (12.4%)	70 (14.9%)	107 (14.0%)
Cardiovascular disease	48 (16.2%)	131 (27.9%)	179 (23.4%)
History or presence of feet wound/ulceration	23 (7.7%)	45 (9.6%)	68 (8.9%)

### Prevalence of Chronic Pain with and without Neuropathic Characteristics in the Lower Limbs

In total, 249 patients reported chronic daily pain for more than three months in the distal lower limbs, giving a prevalence of 32.5% [95% CI 29.2–35.8]. The prevalence of chronic pain was significantly (p<0.01) higher in patients with type 2 diabetes (40.0% [95% CI 35.6–44.4]), than in patients with type 1 diabetes (21.5% [95% CI 16.8–26.2]).

**Figure 1:**
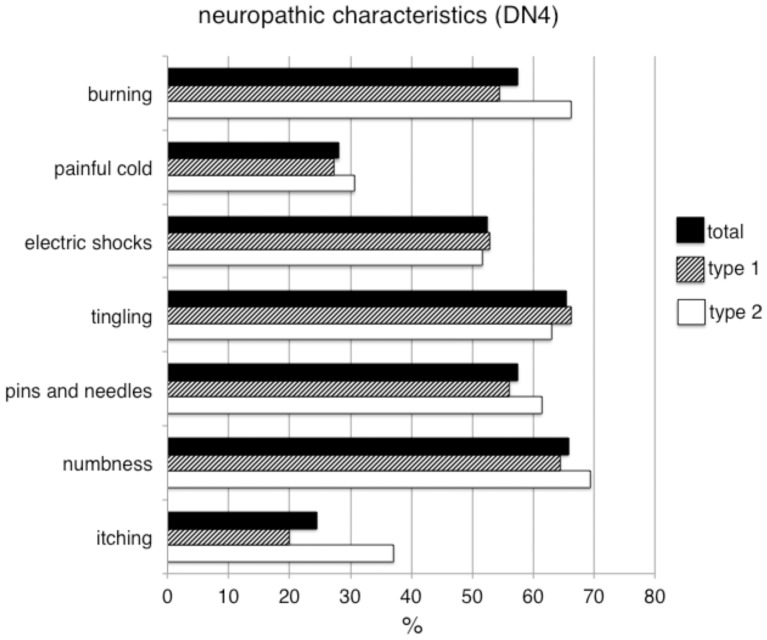
Neuropathic characteristics of pain in diabetic patients. Proportions of patients with type 1 diabetes (white columns), type 2 diabetes (hatched columns) and of the total study population (black columns) reporting the various pain descriptors included in the DN4 questionnaire.

The DN4-interview results (see figure 1) showed that chronic pain had neuropathic characteristics (i.e. DN4-interview score ≥3) in 62.7% (n = 156) of the affected patients. Thus, the overall prevalence of chronic distal lower limb pain with neuropathic characteristics was 20.3% [95% CI 17.4–23.1], and this prevalence was not significantly different between patients with type 1 (14.7% [95% CI 10.7–18.7]) and type 2 (24.7% [95% CI 20.8–28.6]) diabetes.

**Figure 2:**
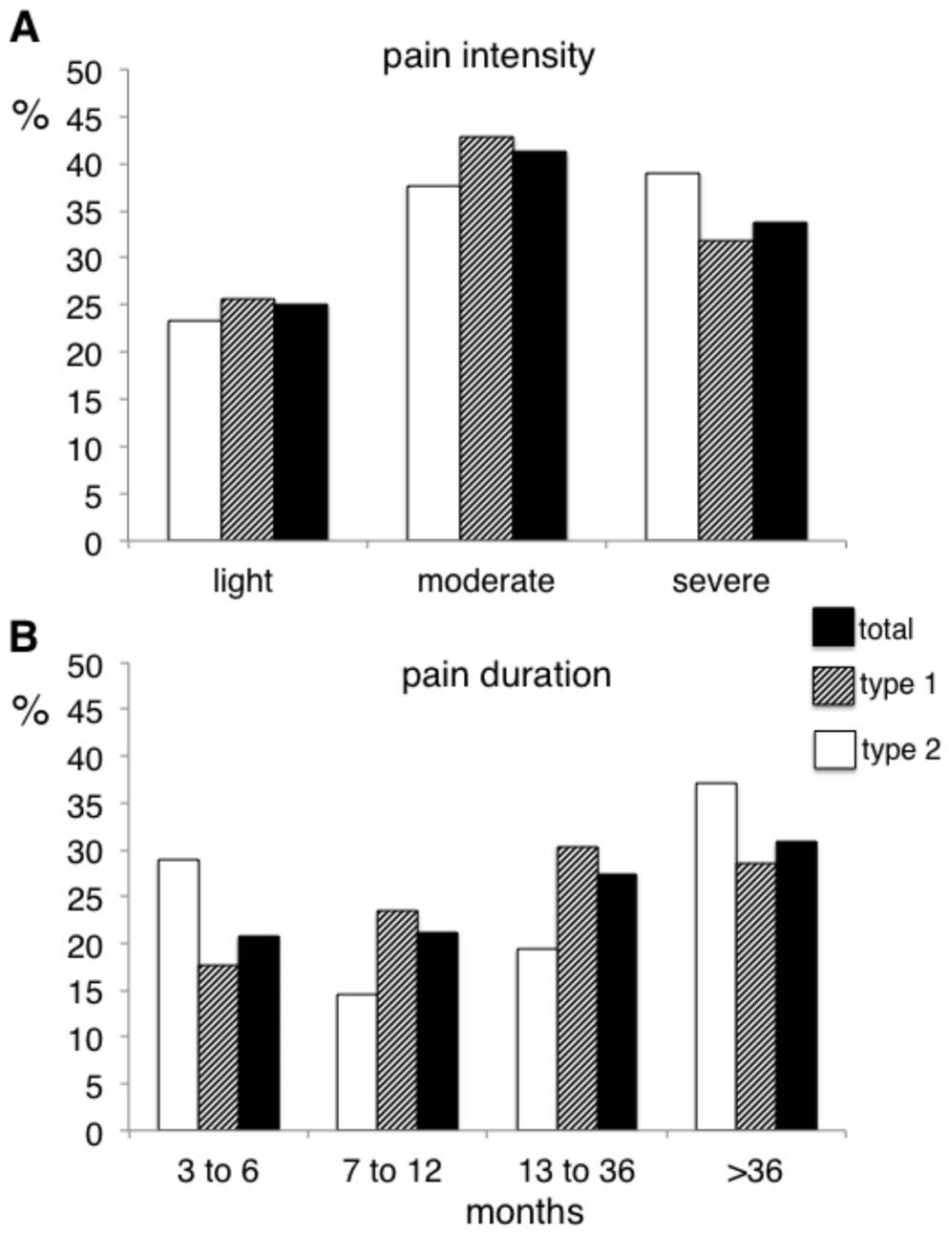
Pain intensity and duration in diabetic patients. A : Proportions of patients with type 1 diabetes (white columns), type 2 diabetes (hatched columns) and from the total study population (black columns) reporting light (numerical rating score from 1 to 3 out of 10), moderate (numerical rating score from 4 to 6 out of 10) or severe (numerical rating score from 7 to 10) average pain intensity over the last week. B : Proportions of patients with type 1 diabetes (white columns), type 2 diabetes (hatched columns) and from the the total study population (black columns), reporting chronic pain for 3 to 6 months, 7 to 12 months, 13 to 36 months and more than 36 months.

Mean pain intensity over the last week was 5.3±2.3 and 76.2% of the patients reported pain of moderate to severe intensity (i.e. NRS score ≥4) (figure 2A). Chronic pain had lasted at least one year in more than half of the patients (57.4%) (figure 2B) and was bilateral (affecting the legs and/or feet) in 74.0% of these patients. Pain intensity was significantly (p<0.001) higher in patients with chronic pain with neuropathic characteristics (6.0±2.1) than in those with chronic pain without neuropathic characteristics (4.3±2.3), and a significantly higher (p<0.001) proportion of patients with neuropathic characteristics had pain of moderate to severe intensity (85.2% vs 61.0%).

The MNSI-examination score obtained suggested the presence of polyneuropathy in 80.1% of the patients with neuropathic characteristics (83.7% of those with type 1 diabetes and 78.8% of those with type 2 diabetes). The proportion of patients with a MNSI-examination score >2 was of 89.5% for patients with bilateral chronic pain. These results, suggesting that distal pain with neuropathic characteristics, particularly when bilateral, is related to polyneuropathy in most patients, were consistent with the investigators’ clinical judgment, with investigators reporting that pain was related to a diabetic polyneuropathy in 91.8% of these patients.

By contrast, only 43.2% of the patients with chronic pain with neuropathic characteristics had an MNSI-questionnaire score suggestive of polyneuropathy (i.e. score ≥7/13), and a similar proportion (48.7%) had an abnormal result for the 10-g monofilament test.

Nearly 20% of the patients with chronic pain with neuropathic characteristics (n = 31), that is 12.4% of the patients with chronic pain, had no neurological signs according to the results of the MNSI-examination and monofilament test.

### Factors Predictive of Neuropathic Pain

Univariate analyses showed that several factors, including older age, type 2 diabetes, the presence of other diabetic complications, a history of foot ulceration, higher total and HDL cholesterol levels, higher triglyceride concentration, a history of alcoholism and a higher BMI, were associated with a higher risk of chronic neuropathic pain in the lower limbs (see table 2). Multivariate analyses identifed the following factors as predictive of neuropathic pain: diabetic nephropathy (OR = 2.59 [95% CI: 1.55–4.32]), a high (>1.6 mmol/l) triglyceride concentration (OR = 2.87 [95% CI: 1.6–5.01] and a history of alcoholism assessed by the consumption of standard drinks (OR = 3.07 [95% CI: 1.41–6.68]).

**Table 2:** Predictive factors of the presence of chronic pain with neuropathic characteristics (univariate analyses).

	chronic neuropathic pain			
	No (n = 595)	Yes n = 156	OR [IC 95%]	p value
Age (years)				0.026
<49.0	147 (86.5%)	23 (13.5%)	1.00	
[49.0; 59.0]	147 (77.8%)	42 (22.2%)	1.83 [1.05;3.19]	
[59.0; 67.0]	149 (80.1%)	37 (19.9%)	1.59 [0.90;2.80]	
≥7.0	152 (73.8%)	54 (26.2%)	2.27 [1.33;3.89]	
Sexe				0.408
Male	331 (80.3%)	81 (19.7%)	1.00	
Female	264 (77.9%)	75 (22.1%)	1.16 [0.82;1.65]	
Type of diabetes				0.001
type 1	250 (85.3%)	43 (14.7%)	1.00	
type 2	344 (75.3%)	113 (24.7%)	1.91 [1.30;2.81]	
HbA1C				0.235
<7%	185 (81.9%)	41 (18.1%)	1.00	
≥7%	408 (78.0%)	115 (22.0%)	1.27 [0.86;1.89]	
Diabetic Retinopathy				<0.001
No	458 (84.0%)	87 (16.0%)	1.00	
Yes	135 (67.2%)	66 (32.8%)	2.57 [1.77;3.74]	
Diabetic Nephropathy				<0.001
No	535 (83.2%)	108 (16.8%)	1.00	
Yes	58 (55.2%)	47 (44.8%)	4.01 [2.59;6.21]	
Cardiovascular comorbidities				<0.001
No	476 (82.5%)	101 (17.5%)	1.00	
Yes	117 (68.0%)	55 (32.0%)	2.22 [1.51;3.26]	
History of foot ulceration				<0.001
No	562 (82.8%)	117 (7.2%)	1.00	
Yes	31 (45.6%)	37 (54.4%)	5.73 [3.42;9.61]	
Total Cholesterol				0.031
<4.1 mmol/L	187 (82.7%)	39 (17.3%)	1.00	
[4.1; 5.0] mmol/L	176 (76.9%)	53 (23.1%)	1.44 [0.91;2.29]	
>5.0 mmol/L	156 (72.2%)	60 (27.8%)	1.84 [1.17;2.91]	
HDL cholesterol				0.018
<1.1 mmol/L	152 (72.0%)	59 (28.0%)	1.00	
[1.1; 1.4] mmol/L	199 (78.7%)	54 (21.3%)	0.70 [0.46;1.07]	
>1.4 mmol/L	185 (83.3%)	37 (16.7%)	0.52 [0.32;0.82]	
Triglycerides				<0.001
<1.0 mmol/L	213 (89.1%)	26 (10.9%)	1.00	
[1.0; 1.6] mmol/L	182 (76.5%)	56 (23.5%)	2.52 [1.52;4.18]	
>1.6 mmol/L	142 (66.7%)	71 (33.3%)	4.10 [2.49;6.73]	
LDL cholesterol				0.477
<2.2 mmol/L	181 (79.4%)	47 (20.6%)	1.00	
[2.2; 2.9] mmol/L	180 (79.6%)	46 (20.4%)	0.98 [0.62;1.55]	
>2.9 mmol/L	172 (75.4%)	56 (24.6%)	1.25 [0.81;1.95]	
High Blood pressure				<0.001
No	277 (85.8%)	46 (14.2%)	1.00	
Yes	312 (73.9%)	110 (26.1%)	2.12 [1.45;3.11]	
Alcoholism				0.004
No	525 (80.9%)	124 (19.1%)	1.00	
Past	23 (59.0%)	16 (41.0%)	2.95 [1.51;5.74]	
Present	45 (73.8%)	16 (26.2%)	1.51 [0.82;2.75]	
BMI				0.074
<25 kg/m^2^	191 (83.4%)	38 (16.6%)	1.00	
[25;30]kg/m^2^	194 (79.5%)	50 (20.5%)	1.30 [0.81;2.07]	
> = 30 kg/m^2^	205 (75.1%)	68 (24.9%)	1.67 [1.07;2.60]	

### Impact of Chronic Pain on Quality of Life, Sleep, Anxiety and Mood

**Figure 3:**
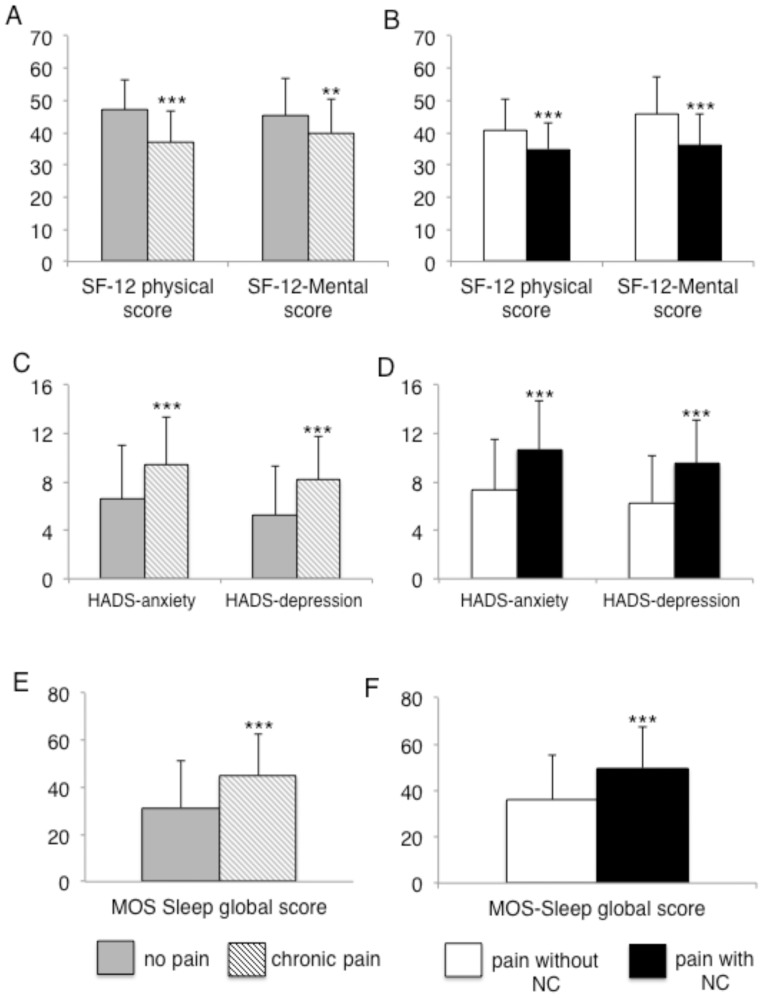
Impact of pain on quality of life, anxiety, depression and sleep in diabetic patients. A : Comparisons of the physical and mental scores of the SF-12 questionnaire between patients with (gray columns) and without (hatched columns) chronic pain and B : between patients with chronic pain with (white columns) and without neuropathic characteristics (black columns). C : Comparisons of the anxiety and depression HADS scores between patients with (gray columns) or without (hatched columns) chronic pain and D : between patients with chronic pain with (white columns) and without neuropathic characteristics (black columns). E : Comparisons of the MOS-Sleep score between patients with (gray columns) and without (hatched columns) chronic pain and F : between patients with chronic pain with (white columns) and without neuropathic characteristics (NC) (black columns). **p<0.01; ***p<0.001.

Patients with chronic pain had significantly lower physical and mental health subscores for the SF-12, greater impairment of sleep quality and higher anxiety and depression scores (see figure 3A, C, E). Patients with chronic pain with neuropathic characteristics had a significantly lower quality of life, more sleep problems and higher anxiety and depression scores than those with chronic pain without neuropathic characteristics (figure 3B, D, F).

The multivariate analyses showed that:

- The independent factors predictive of a lower physical component of quality of life (SF-12 questionnaire) were: being female (OR = 2.33 [95% CI: 1.18–4.56]), a pain intensity>4 (OR = 5.94 [95% CI: 2.43–14.48]), the presence of foot wound/ulceration (OR = 6.76 [95% CI: 2.33–20.48]), a HbA_1c_ level ≥7% (OR = 2.01 [95% CI: 1.01–4.02]) and a BMI ≥30 kg/m^2^ (OR = 2.53 [95% CI: 1.38–5.62]).
- The independent factors predictive of a lower mental component of quality of life (SF-12 questionnaire) were: being female gender (OR = 2.62 [95% CI: 1.46–4.67]), a DN4 score ≥3 (OR = 3.81 [95% CI: 2.17–6.68]) and a triglyceride concentration >1.6 mmol/l (OR = 2.02 [95% CI: 0.94–4.29]).
- The independent factors predictive of a lower quality of sleep were: a DN4 score ≥3 (OR = 3.22 [95% CI: 1.71–6.06]), a pain intensity >4 (OR = 4.47 [95% CI: 1.98–10.12]) and an age <49 years (OR = 5.30 [95% CI: 1.90–13.70]).
- The independent factors predictive of a higher score for anxiety (HADS-A) were: a DN4 score ≥3 (OR = 3.2 [95% CI: 1.72–5.96]), a pain intensity >4 (OR = 2.3 [95% CI: 1.06–4.89]) and a triglyceride concentration >1.6 mmol/L (OR = 3.87 [95% CI: 1.82–8.20]).
- The independent factors predictive of a higher score for depression (HADS-D) were: a DN4 score ≥3 (OR = 4.38 [95% CI: 2.27–8.47]), a BMI ≥30 kg/m^2^ (OR = 1.94 [95% CI: 0.98–3.82]) and a MNSI-questionnaire score ≥7 (OR = 2.20 [95% CI: 1.15–4.13]).

### Treatment of Neuropathic Pain

About three quarters (72.7%) of the patients with chronic pain with neuropathic characteristics had previously consulted for their pain. Most had consulted a general practitioner (69.9%) or a diabetologist (65.6%), with smaller proportions consulting a neurologist (26.6%), a rheumatologist (16.8%) or a pain specialist (4.6%).

More than two thirds (68.9%) of the patients with neuropathic pain received treatment for their pain. However, only 38.6% of these patients were treated with at least one of the recommended first-line treatment: antiepileptic drugs (24.5%) and/or antidepressants (16.9%).

## Discussion

This cross-sectional observational study, the largest to date on this topic to be carried out in France, indicate that one in five diabetic patients suffers daily pain with neuropathic characteristics, in the legs and/or feet, over periods of at least three months. Our data also show that chronic pain with neuropathic characteristics has a major impact on quality of life, mood, anxiety and sleep and that only a minority of patients receive one of the recommended first-line treatments for neuropathic pain. Finally, our finding suggest that screening tools for neuropathic pain such as the DN4 questionnaire, may be of value for the systematic screening of diabetic patients for polyneuropathy.

Our overall estimate of the prevalence of distal neuropathic pain in diabetic patients is consistent with previously reported values of the prevalence of painful diabetic polyneuropathy (PDPN), despite the use of different diagnostic criteria in different studies. Three studies carried out in community-based samples in the UK provided estimates of 16.2% [23]), 26.4% [24] and 21.0% [25]. A structured interview and standardized examination were used in two studies on relatively small cohorts of patients [23], [24], whereas only questionnaires – the neuropathy symptom score (NSS) and the neuropathy disability score (NDS) – were used in a much larger cohort of patients receiving community-based health care [25].

In general, our approach, based on validated questionnaires and standardized neurological examination (i.e. MNSI and the monofilament test), was similar to that used in these studies. However, we gave priority to the patients’ symptoms (the presence of chronic pain, defined as daily pain for at least 3 months, and neuropathic characteristics) in our case definition. Neuropathic pain has been shown to have particular clinical characteristics that can be identified with simple, easy-to-use clinical tools based on the analysis of the terms used by the patients to describe their pain (i.e. pain descriptors) [10]. In our opinion, the use of specific neuropathic screening tools could increase diagnostic accuracy for PDPN, which is, by definition, neuropathic [6]. This approach might make possible to decrease the number of false positives, because pain in the lower limbs of diabetic patient may not necessarily be related to polyneuropathy, even in the presence of signs of neuropathy. For example, a patient with polyneuropathy may suffer pain related to ischemia, which is not neuropathic and requires specific management. Conversely, this approach may also make it possible to decrease the number of false negatives, because some patients may have symptoms suggestive of polyneuropathy without obvious neurological signs on bedside examination. Consistent with this notion, our data confirm that the prevalence of neuropathic symptoms in the lower limbs was higher than that of neurological signs in patients with diabetes and chronic distal pain. In our study, about 20% of the patients with distal neuropathic pain had no neurological signs, corresponding to about 12% of those with chronic pain. Similar results were reported by Abbott et al. [25] who found that 32% of their patients had symptoms of neuropathy, whereas only 20% also had neurological signs. Thus, screening for polyneuropathy on the basis of the presence of sensory motor deficits alone may result in an underestimation of the proportion of diabetic patients with a polyneuropathy and to the undertreatment of these patients. The use of standardized clinical examination, such as the MNSI-examination and monofilament test, also result in the underdiagnosis of diabetic polyneuropathy because these tests do not assess the functionality of small nerve fibers which can be preferentially or selectively injured in diabetic patients, especially in patients with neuropathic pain [4]. Thus, our data support a more systematic use of tests [26] or clinical scales [27], which have been more specifically developed for the identification of small fiber neuropathy.

A few other epidemiological surveys in diabetic patients have used screening tools for neuropathic pain. Two studies carried out in the Middle East Area and based on the DN4 questionnaire reported higher prevalence rates than in our study. In one study carried out Saudi Arabia [28] on a cohort of 1039 patients, the prevalence of pain with neuropathic characteristics was of 53.7%, but the site and duration of neuropathic pain were not reported in this study. The prevalence of chronic neuropathic pain was, therefore, probably much lower. This conclusion is supported by the results of another study carried out in several countries of the Middle East [29], which reported an overall prevalence of pain with neuropathic characteristics of 65.3%, but a prevalence of 38.0% for pain with a duration of at least one year. In another study in specialized centers in Belgium including more than 1100 type 1 or type 2 diabetic patients [13], a combination of the DN4 questionnaire and the monofilament test was used for PDPN diagnosis, giving in a prevalence of 14.1%. This relatively low prevalence, may reflect the low sensitivity of the monofilament test for the diagnosis of polyneuropathy, particularly when this condition preferentially affects the small fibers, as this test assesses only the function of large fibers. A similar prevalence of PDPN of 16.0% was reported in a study from Turkey combining nerve conduction studies and the use of another neuropathic pain screening tool, the Leeds Assessment of Neuropathic Symptoms and Signs (LANSS) [30]. However, it is also likely that patients with a small-fiber in this study went undetected in the nerve conduction studies. However, despite the use of variable diagnostic criteria, our data concur with those of previous studies to confirm that chronic neuropathic pain is a major complication of diabetes concerning 15 to 25% of the patients in Western Europe and probably a even higher proportion of patients (at least 30%) in the Middle East.

The main risk factors for chronic neuropathic pain identified here are similar to those previously reported for painful diabetic neuropathy [13] and for diabetic neuropathy in general [1], [3]–[5]. They include older age, type 2 diabetes mellitus, the presence of other diabetic complications and the presence of metabolic syndrome, again highlithing the potential role of microvascular alterations in the mechanisms underlying both painful or pain-free diabetic neuropathy [31], [32].

Relatively few studies have specifically assessed the impact of neuropathic pain on the health-related quality of life (QoL) and psychological comorbid conditions of diabetic patients [13]–[16]. Consistent with the results of Van Acker et al. [13], our data indicate that the chronic neuropathic pain associated with diabetes is severe and has a major impact on QoL. More than half of our patients reported pain lasting for more than one year, and almost three quarters of them had pain of at least moderate intensity (≥4 of maximum score of 10). Both the mental and physical components of QoL, as measured with the SF-12 questionnaire, were significantly more impaired in patients with chronic pain than in those without pain. Patients with chronic pain also had significantly more depression, anxiety and sleep disorders. Interestingly, the frequency of these impairments was significantly higher in patients with chronic pain with neuropathic characteristics than in those with chronic pain without neuropathic characteristics. The specific impact of the neuropathic characteristics of chronic pain on QoL and psychological comorbid conditions was further confirmed by the multivariate analyses, which showed that DN4 score (reflecting the neuropathic nature of pain) was an independent predictive factor for QoL impairment, high anxiety and depression scores and sleep disturbances in patients with chronic pain. Similar results were reported in two recent large epidemiological surveys in the general population [12], [33]. This specific impact of neuropathic pain may reflect its specific pathophysiological mechanisms, but also the poor recognition of this type of pain, resulting in inadequate treatment. Consistent with this hypothesis, we found that although 72% of our patients had consulted for their pain, fewer than 40% had received one of the recommended first-line treatment for neuropathic pain [34], [35]. Similar results have been reported before [13], [24], indicating that painful diabetic neuropathy is largely undertreated or inadequately treated in several countries, even in a specialized setting.

The unmet needs regarding the management of neuropathic pain, which are not specific to diabetic patients [12], may reflect the underdiagnosis of this type of pain. Thus, as suggested in some recent reviews and guidelines [2], [4], [5], neuropathic pain should probably be specifically assessed in diabetic patients. Neuropathic pain screening tools might be useful for this purpose. Consistent with the results of a recent study [36], our data showed strong concordance between the DN4 and MNSI results (up to 89% in patients with bilateral neuropathic pain) and between the DN4 results and the clinical judgement of the investigators, suggesting that screening for neuropathic pain could also improve the diagnosis of polyneuropathy in diabetic patients. By contrast, the relatively poor concordance between the results of the DN4 questionnaire and the monofilament test suggests that this test, which evaluate protective tactile/pressure sensation and is often used for the prevention of diabetic foot, is not sensitive enough for the identification of painful diabetic polyneuropathy.
